# Citizen science approach to assessing patient perception of MRI with flexible radiofrequency coils

**DOI:** 10.1038/s41598-024-53364-x

**Published:** 2024-02-02

**Authors:** Lena Nohava, Raphaela Czerny, Martin Tik, Dagmar Wurzer, Elmar Laistler, Roberta Frass-Kriegl

**Affiliations:** 1 High Field MR Center, Center for Medical Physics and Biomedical Engineering, Medical University of Vienna, Vienna, Austria; 2 Bundes(real)gymnasium BG/BRG Keimgasse, Mödling, Austria

**Keywords:** Biomedical engineering, Clinical trials

## Abstract

Magnetic Resonance Imaging (MRI) is a major medical imaging modality, which is non-invasive and provides unique soft tissue contrast without ionizing radiation. The successful completion of MRI exams critically depends on patient compliance, and, thus patient comfort. The design, appearance and usability of local MRI radiofrequency (RF) coils potentially influences the patients’ perception of the exam. However, systematic investigations and empirical evidence for these aspects are missing. A questionnaire specifically evaluating the impact of RF coils on patient comfort in MRI would be a valuable addition to clinical studies comparing the performance of novel flexible RF coils with standard rigid coils. This paper describes the development of such a questionnaire in the scope of a citizen science (CS) initiative conducted with a group of students at the upper secondary school level. In this work, the CS initiative is presented in the format of a case report and its impact on scientific projects and the students’ education is outlined. The resulting questionnaire is made available in German and English so as to be directly applicable by researchers working on the clinical evaluation of novel RF coils or the comfort evaluation of specific hardware setups in general.

## Introduction

Magnetic resonance imaging (MRI) is one of the most important imaging modalities in modern medical diagnostics. MRI offers excellent soft tissue contrast and is suitable for high-resolution imaging of the structure and function of various tissues and organs in the body without ionizing radiation.

Although MRI is a non-invasive imaging modality, patients may perceive the examination as frightening and/or uncomfortable^1–4^. The causes of discomfort before or during MRI examinations are complex and may be related to spatial confinement (claustrophobia), acoustic noise or vibrations, ambient temperature, the examination duration (5 min to 1 h on average depending on the body region), uncomfortable positioning (e.g., “superman position”, i.e. prone position, face down on the MRI table with arms extended above the head) or cushioning materials and fixations to minimize patient motion, as well as individual anxiety levels, among other factors^3,5^. In addition, dizziness, nausea, peripheral nerve stimulation, and a metallic taste in the mouth have been reported with high and ultra-high field systems^6–11^.

It is noteworthy that patient compliance is of major relevance for the successful completion of MRI exams. Patients who feel anxious or uncomfortable during the imaging procedure are more likely to terminate the examination before completion or to move during image acquisition causing image artifacts^3,12–14^. This entails the necessity to repeat the scan or leads to unexploitable images, ultimately resulting in delayed diagnosis and treatment, as well as significant costs in terms of time and resources for healthcare organizations^1,15,16^.

Several pharmacological and non-pharmacological interventions to address anxiety issues have been proposed^17^, including recommendations for scanner hardware and imaging protocols^18,19^. Among the most promising non-pharmacological interventions are video demonstrations, telephone conversations with radiographers^12^, cognitive-behavioral therapy^20^, and virtual reality simulations^21^.

Radiofrequency (RF) coils are essential hardware components in MRI used for generating and receiving the MR signal. The positioning of RF coils is a crucial aspect of the examination process, which ideally involves experienced technical personnel, a compliant patient and an application-specific coil placed as close as possible to the body region of interest. Mechanically rigid coils of fixed size only partially meet the need for a coil that fits the body shape of each patient, resulting in poor image quality in some cases. In contrast, mechanically flexible RF coils offer an attractive alternative, especially for body regions that vary strongly in size and shape within the patient population. The development of such flexible coils is subject of ongoing research, successfully demonstrating that the detection performance is comparable or better than with rigid coils, as reported in examples from literature^22–27^ and summarized in a recent review article^28^.

While previous investigations on flexible RF coils focus primarily on technological parameters, in terms of either technical feasibility or performance, the use of flexible instead of rigid coils could also positively influence the subjective perception of the patient during the measurement. Flexible coils could allow for a more ergonomic positioning of patient and coil in the scanner and help to avoid pressure points, especially for body parts of variable size. In the particular case of breast MRI, flexible coils have been shown to facilitate supine instead of typical prone patient positioning^26,29^, which has great potential to expand the examinable patient population to women who have received radiation therapy and cannot lie prone due to complications like edemas or fractures. In addition, flexible coils typically have a slim and lightweight design, which could make them less intimidating and potentially put less mechanical pressure on the patient’s body. Furthermore, it is often suggested that the measurement setup could be shortened due to the potentially easier handling of flexible coils^28^, leading to reduced overall examination times and thereby increasing patient throughput. However, a systematic investigation and empirical evidence for the promoted benefits of increased comfort and simplified handling is missing.

For this reason, it would be beneficial to develop a patient questionnaire dedicated to investigating the RF coil’s impact on comfort in MRI. It could be used as an add-on for clinical studies evaluating the performance of novel flexible RF coils in comparison to rigid standard coils. While prior works using questionnaires to assess patient comfort during MRI mainly evaluated anxiety either with standardized anxiety scales^3,20,30^, or using custom forms^21,31^, none of them is explicitly focused on the impact of the RF coil.

This article describes the process of developing such a questionnaire in the scope of an educational citizen science (CS) initiative. CS, designating the public participation in scientific research or known as “community science”, involves citizens, i.e., members of the general public, in any aspect of authentic research projects^32^. Here, students at the upper secondary level were recruited and invited to (1) fill out a preliminary version of the questionnaire after a series of activities to familiarize with MRI, and (2) provide active feedback and suggestions on it. The main expected benefit of this approach is a questionnaire truly tailored to the MRI experience and knowledge level of the study participants. Citizen scientists usually either know MRI examinations only from the patient's point of view or are completely inexperienced in this field, which enables them to much better put themselves in the position of the patients participating in clinical studies in comparison to experienced MRI researchers and, thus, to decisively improve the study design (i.e., the questionnaire).

On top of that, the CS approach provides the opportunity to make academic research more accessible to the general public, which is considered increasingly important by the scientific community, especially in the STEM domain, i.e. Science, Technology, Engineering and Mathematics, e.g. Ref.^33^. This work aims at increasing the knowledge of the participating citizen scientists about health care technology and at positively influencing their attitude towards science in general. Therefore, both, improving the scientific outcome of the clinical study and generating added value for the citizen scientists represent major objectives of the described CS initiative.

To the authors’ knowledge, the outlined CS initiative is unprecedented in the field of MRI hardware development. In this article, the employed CS approach is delineated in detail, the developed questionnaire is presented, and the research team consisting of full-time MRI scientists shares insights and lessons learned from this first CS experience in a format comparable to a case report.

## Results

### CS in the frame of MRI RF coil development

**Figure 1:**
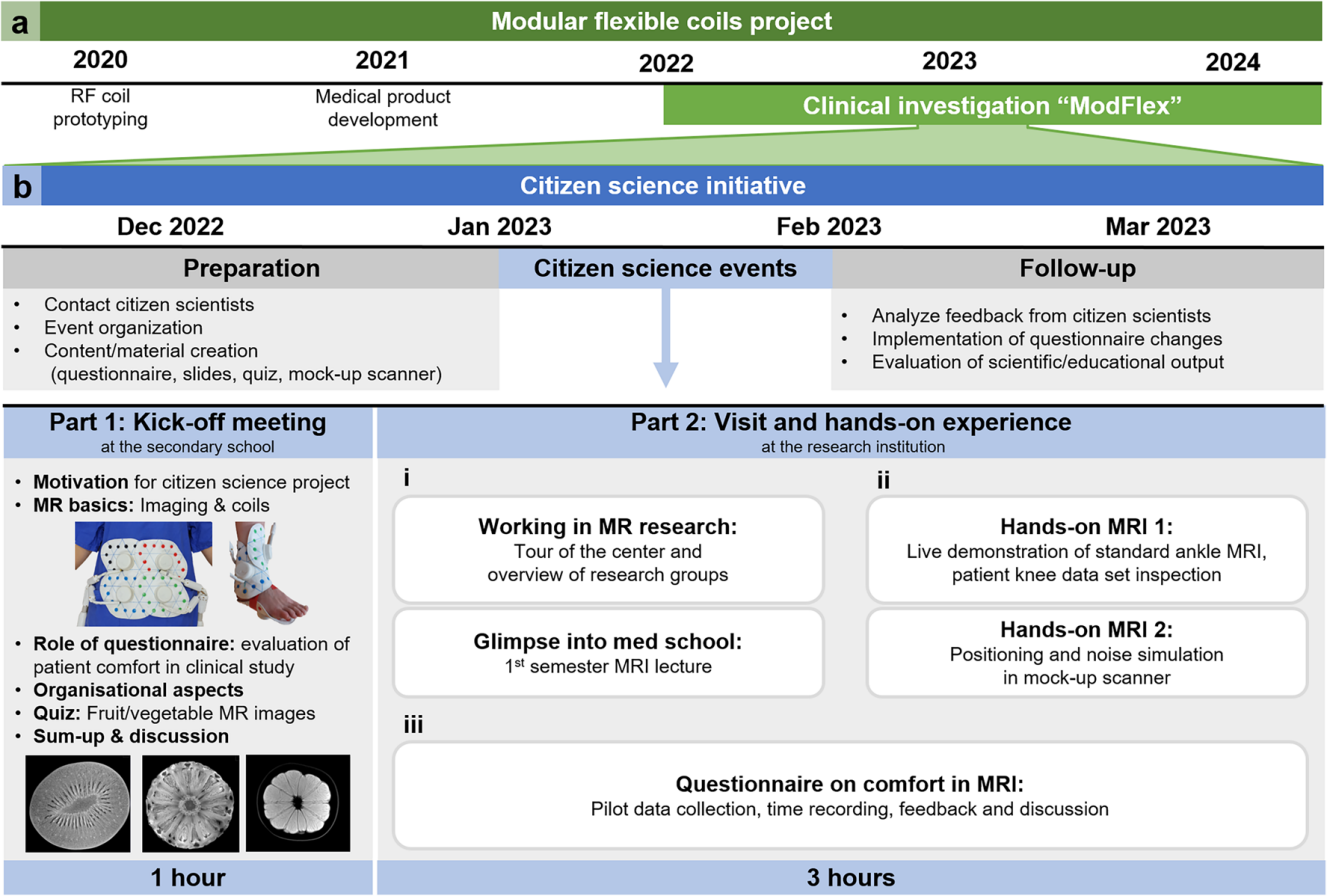
Timeline of clinical study context and organization of the CS initiative.

The presented CS initiative is part of a large-scale research project targeting the development and evaluation of novel modular, flexible RF coils. In particular, a vest-like RF array for breast imaging^26^, and a modular system of RF arrays to be used on the neck, ankle, spine and hip^27^. In Fig. 1a, a general overview of the research project is given and the relation of the CS part to the overall project context and organization is specified. After the development of coil prototypes and their implementation as clinically applicable devices, the project is currently at the stage of evaluating their imaging performance in comparison to standard clinical reference coils in a clinical investigation, i.e. the “ModFlex” study (see Methods section for details). The interventions in this study consist of two MRI exams, one using a novel flexible RF coil and one using the standard reference product. To additionally evaluate patient comfort, a questionnaire to assess the physical and psychological well-being before, during and after the exam will be filled out by the study participants after each exam, so as to enable a comparative analysis. The development and refinement of this questionnaire is the main focus of this work and was performed in two steps: First, an initial (i.e., pre-CS) version was proposed by the researchers involved in the “ModFlex” study as described in the Methods section. Second, this questionnaire was revised and refined in collaboration with citizen scientists.

### Organization and materials of the CS approach

The citizen scientists who participated in the presented initiative are 24 students of the upper secondary school level (15–17 years old, 12 male and 12 female), who are enrolled in so-called “model classes” for the promotion of particularly talented students. The group consisted of a whole class in the 11th grade and, in addition, several interested students in the 10th grade.

**Figure 2:**
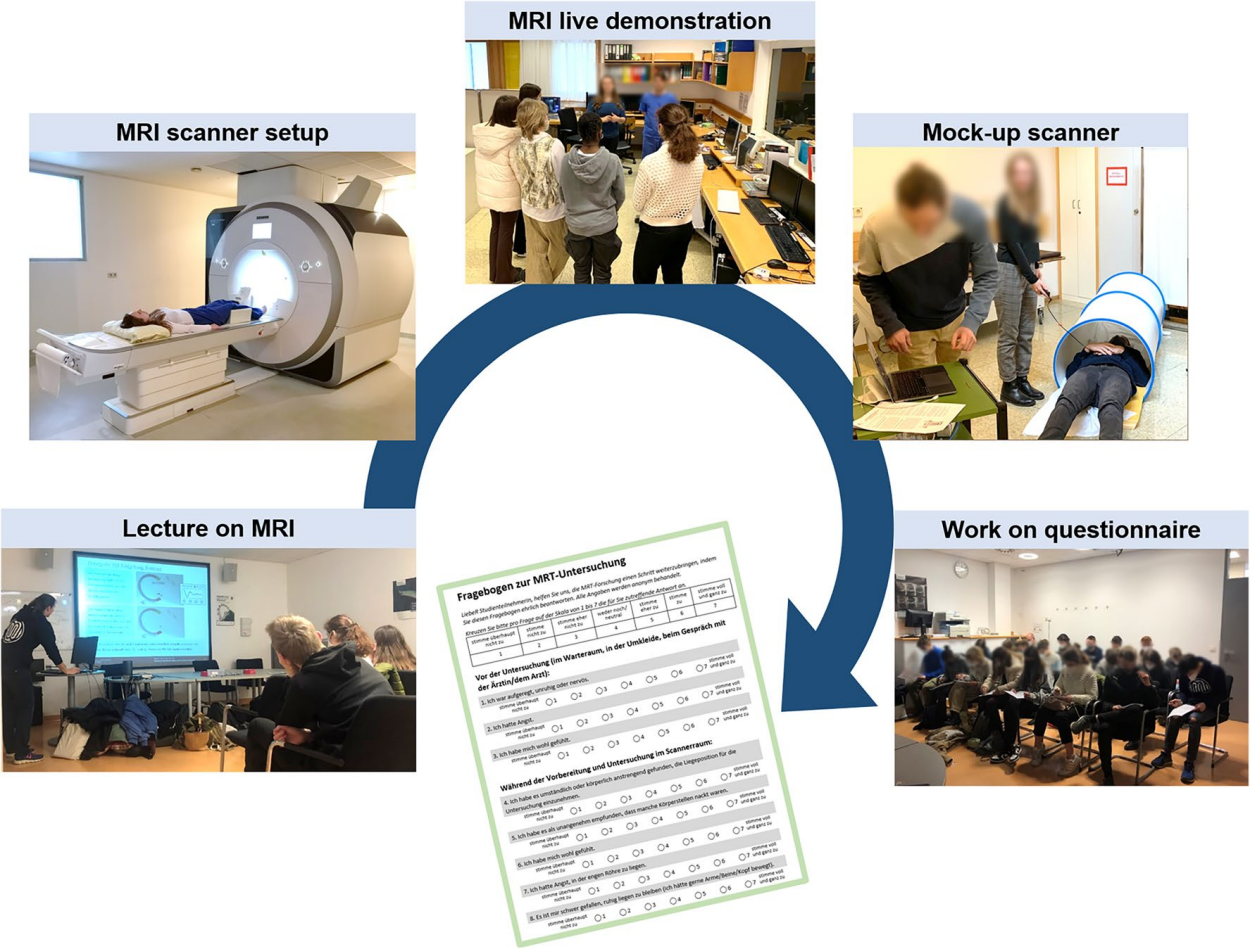
Graphical summary of the different activities offered to citizen scientists during their visit at the research institution.

Figure 1b shows the detailed organization and implementation of the CS initiative composed of a preparation phase, the actual CS events (i.e., kick-off meeting and student visit to the research institution) and a follow-up phase. The preparation of the CS events started with selecting and contacting the participating citizen scientists, laying out the event schedule and creating the required material, i.e., the pre-CS version of the questionnaire, digital slides for introductory presentations, and a mock-up MR scanner (see Fig. 2 and description below).

The kick-off meeting established the initial contact between the research team and citizen scientists in their school. The researchers explained the motivation for the project in general, introduced the students to the basics of MRI, e.g. the components needed to perform MR imaging, and illustrated the function and different types of RF coils. A key aspect was to emphasize the important role of the questionnaire for the outcome of a genuine clinical study. Further, organizational aspects were clarified, for example the planned program during the students’ MR center visit. The kick-off meeting finished with an interactive quiz in two groups, where students got the task to guess which fruit or vegetable was displayed in a series of MR images. This activity served as an icebreaker but also allowed a first introduction to possible image contrasts in MRI and demonstrated the complexity of MR image interpretation.

The second CS event was a visit to the MR center. Due to the infrastructure of the research center and safety requirements, the students had to be divided into two groups of 12. The two groups alternatingly participated in two types of activities that can be described as “informative” (see Fig. 1b, Part 2: i) and “hands-on” (see Fig. 1b, Part 2: ii), with details given below. Subsequently, work on the questionnaire (see Fig. 1b, Part 2: iii) was done with the whole group reunited in a seminar room. The activities proposed during the CS event at the research institution are summarized with photographs in Fig. 2.

“Informative” agenda items (Fig. 1b, Part 2: i) aimed at making the CS event particularly valuable for the participating students by providing exclusive insights into daily research work and the dissemination of basic knowledge about state-of-the-art MRI technology. To this end, a guided tour of the MR center was offered showing the students the work of different research groups, with stops at the high field (3 T) and ultra-high field (7 T) MRI scanners, the technical cabinets and the RF coil laboratory and workshop. Additional personnel were on site to supervise them around delicate equipment. Next, the students got a “glimpse into med school” with a lecture on MRI at 1^st^ semester university level. They were also informed about the possibility to apply for summer internships at the research institution.

“Hands-on” agenda items (Fig. 1b, Part 2: ii) focused on optimally preparing the citizen scientists for their work on the questionnaire. The students could actively experience what it would feel like to undergo an MRI examination and acquire MR images. Therefore, a live demonstration of a volunteer undergoing an ankle MRI was given at the 3 T scanner. The demonstration started with a short explanation of the information patients and volunteers receive before a study exam, followed by the setup preparation inside the scanner room using a standard rigid ankle coil. During image acquisition at the main scanner console, students witnessed MRI sequence noise and the procedure to acquire images with multiple contrasts in different imaging planes. On a satellite console, students had the possibility to inspect knee MR images of an anonymized patient data set with bilateral cysts. With the help of an online knee anatomy explanation^34^ displayed on a laptop they could try to identify anatomical details, as for example the cruciate ligament. With the help of a mock-up MRI scanner, i.e. a plastic bore imitating crucial aspects of the MRI environment (e.g., limited space, light conditions) while obviating the risks associated with high electromagnetic fields, and headphones with an audio playback mimicking MRI sequence noises, each student got to simulate an MRI exam for one to two minutes. All agenda items contributed to their better understanding of MRI in general and the patient experience during an MRI exam, important for the ultimate goal of evaluating the questionnaire.

The final activity of the CS event was to test, review and revise the questionnaire in plenum (Fig. 1b, Part 2: iii). The students filled out printed pre-CS questionnaires, which was followed by an interactive discussion. This session was moderated by the research team to guide students and cover different topics of discussion (e.g. how they felt during the mockup scanner experience, what they thought of the questionnaire’s content, formatting etc.). For each feedback students brought up, the researchers asked for concrete suggestions on how to improve the questionnaire. Additionally, an optional electronic newsletter informing about the future progress of the research project was offered.

### Scientific outcome

**Figure 3:**
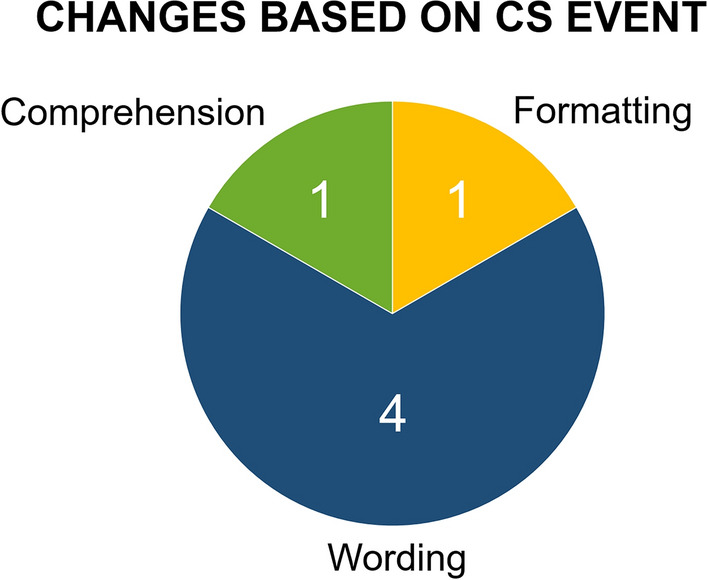
Type of changes suggested by the students and implemented in the final version of the patient comfort questionnaire. 6 changes in total (applied to 20 items and a short introduction).

The length of the pre-CS version of the questionnaire consisting of 20 questions was validated by the CS event, as it took the students between two and four minutes to complete it, which is below the upper limit of five minutes defined by the research team conducting the “ModFlex” study. During the interactive discussion session, nine potential issues in the questionnaire were brought up by the students also proposing direct adaptations. Based on these suggestions, six changes were actually implemented concerning the formatting of the response scale, part of the introductory text, and four out of 20 items: For the detailed response scale, which is shown on each page above the first question item, the boxes to tick were eliminated to avoid confusion. In the introduction, unnecessary technical terms were removed. The last item, asking for further remarks, was rephrased to a direct question to encourage participation. The other adaptations included changes in wording to avoid misunderstandings in items no. 5, 8, and 18. Figure 3 shows the different categories of implemented changes. The resulting version of the questionnaire in German (i.e., official language in the country where the study is conducted) can be found in Supplementary Data S1, Supplementary Data S2 contains the corresponding English translation. This questionnaire can be directly used in the “ModFlex” study and can serve as a basis for other future clinical investigations to evaluate the influence of the RF coil design on patient comfort.

**Figure 4:**
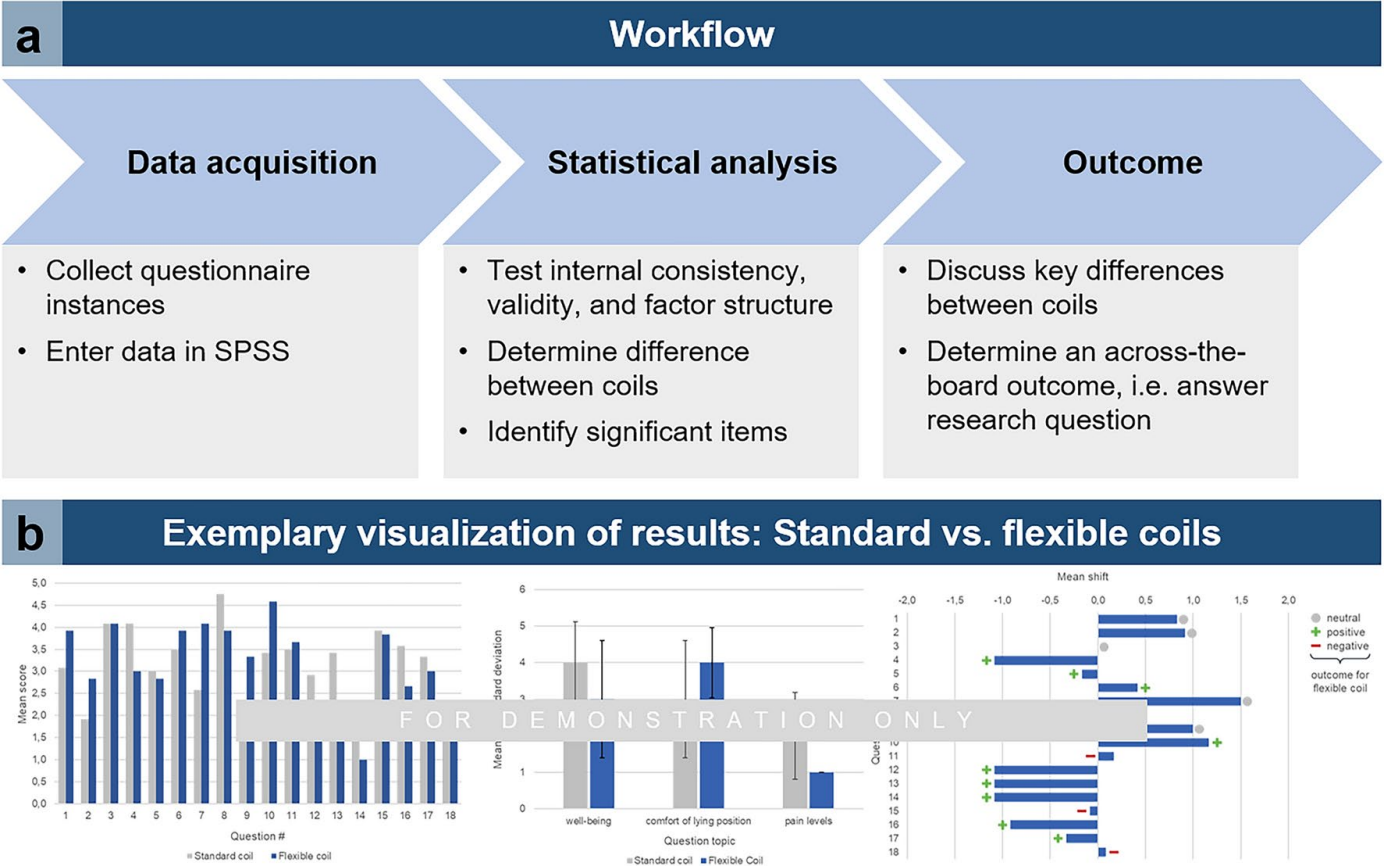
Learnings concerning the evaluation of patient comfort derived from the CS initiative. (**a**) Data analysis workflow and (**b**) exemplary visualization of results comparing two RF coils using pilot data: Mean score per question (excluding open-ended questions), detailed results of three essential questionnaire items and mean absolute shift between measurements per item with additional information whether the result is a positive or negative outcome for the novel flexible coils.

Additionally, organizing and conducting the presented CS events made the research team more experienced in the acquisition and analysis of survey data and helped to set up and optimize the workflow of integrating the questionnaire into the clinical study. Thereby, the following strategy was defined: The questionnaire was formatted so as to fit on two pages, to be printed out on a single sheet of paper that can be handed out to participants for data collection. A digital questionnaire was omitted, since different departments are involved in the clinical study and additional hardware would be needed to facilitate digital data acquisition. A randomized chronological order of measurement sessions for each subject with either the flexible or standard coil will be implemented to avoid bias. Regarding the personnel conducting the questionnaire study, it was determined that having the same individual consistently manage patient or volunteer interactions would be the most effective approach in order to minimize examiner bias, which occurs when the individual administering the questionnaire influences the participants' responses. The acquired questionnaire data will be manually transferred to a statistics software for further evaluation (in this case: IBM SPSS Statistics, IBM, Armonk, NY, USA). The pilot data produced by the students when filling out the pre-CS questionnaires was used to set up the statistical analysis and visualization of the results. This comprises statistical hypothesis testing to determine significant differences between the measurement groups. An overview of the derived workflow can be seen in Fig. 4, explaining the data acquisition and subsequent statistical analysis to formulate an outcome for the comparison of the novel flexible RF coils to the standard rigid coils using the questionnaire. Exemplary visualizations of results using pilot data are shown for demonstration purposes.

### Educational outcome

The presented CS initiative contained a series of activities that aimed at generating added value for the involved citizen scientists. In this regard, the following highlights and lessons learned have been identified in closing discussions with the involved teachers. Note that this educational outcome is described only informally and qualitatively. A systematic quantitative analysis of the effects of the CS events on the students’ education and general opinion about science was not targeted in the presented work and would require a more elaborate study design and larger sample size.

In particular, the integration of the CS initiative into the school environment was well received by students and teachers, as this kind of work exceeds typical curricular activities. The short-term CS events could be easily scheduled in accordance with regular schooling, and the organizational burden for the teachers was moderate. However, a better knowledge transfer, e.g. about healthcare technology, could have been achieved via the long-term involvement of the students in scientific activities and the perennial integration of the CS initiative into the school curriculum. For teachers and researchers, this would be associated with an increased management effort, especially concerning the scheduling of multiple CS events over the years and the preparation of a greater amount of material dedicated to teachers as well as students.

In closing discussions, the teachers also reported that the presented CS initiative positively impacted the students’ perception of science in general, and that some students show greater interest in research than before the CS events. Three students from the CS group, which is the maximum number of available positions, will participate in a four-week summer internship at the research institution. Didactically, the described stepwise approach of introducing the students to scientific work was considered important to generate profound interest: (1) The kick-off meeting facilitated the first contact between students and scientists in an environment well known to the students. This way, the kick-off meeting served as an icebreaker helping to maximize the efficiency of the excursion. (2) During the students’ visit at the research institution, they had the chance to explore a scientific work environment and to have open discussions with senior and junior researchers together with their classmates. (3) Summer internships provide some students with the opportunity to dive deeper into scientific work at an individual level. According to the teachers’ opinion, this stepwise approach was especially important for female students, who benefitted from an increased confidence and the possibility to familiarize with the social environment before actually applying for an internship in science.

## Discussion

In this work, the development of a questionnaire dedicated to investigating the RF coil’s impact on patient comfort in MRI using a CS approach is delineated.

The resulting questionnaire can be directly used in the clinical “ModFlex” study. Since the number of remaining subjects in the envisioned study population is high, statistically significant outcomes can be expected. Furthermore, the proposed questionnaire can be used by other research groups as an add-on for clinical studies evaluating RF coil performance and, in principle also for the comfort evaluation of various (two or more) hardware setups in MRI, e.g., motion tracking devices. Depending on the studied hardware setups, this might require minor adaptations of some questionnaire items. In all cases, the questionnaire is designed for comparative studies and needs to be filled out after each individual measurement.

The absolute number of changes implemented in the questionnaire resulting from the CS initiative may not seem high, but it should be considered that the questionnaire had already been critically revised by experienced researchers before handing them out to students. The authors are confident that even minor changes can lead to a better understanding of the questionnaire and as a consequence minimize the time to fill it in, which in turn could improve patient compliance and limit non-response bias and dropout rate.

During questionnaire development and testing, the research team became aware of confounding factors that could have an undesired impact on the study outcome. Factors that are difficult to correct for were identified, e.g. that patients can inevitably identify the newly developed coil either by common sense or by carefully reading the patient information forms they obtain before their study participation. This could lead to some sort of “pain placebo” effect, where participants may perceive less pain because they trust the new device to be superior to the current standard or that they perceive increased pain or anxiety because they consider the new coil as too experimental and as a potential safety hazard. In addition, the different infrastructure at the research-oriented MR center with a subjectively more open and welcoming atmosphere compared to the hospital infrastructure where the breast patient measurements are performed could alter the perception of comfort. Some patients scheduled for breast cancer imaging get their preliminary diagnosis immediately before the MRI measurement or have a biopsy scheduled directly afterwards which can evidently also impact their perception of the MRI exam and experienced comfort. This latter factor could be corrected for by investigating whether they have any other medical treatments or consultations scheduled on the same day.

The pilot data collected with the students only enabled a very preliminary planning of the data analysis and visualization workflow with 12 artificially assembled paired samples. Real patient data sets will be required to get more concrete in determining suitable analysis tools and optimize or eventually even add some questionnaire items. For this purpose, it will be indispensable to perform an interim analysis, e.g. at 25% and 50% recruitment status. The collected data will be analyzed using paired t-tests if the data distribution meets the assumptions required for this test (i.e., normal distribution, homoscedasticity, continuous data). If the data distribution does not fit the criteria for the t-test, nonparametric alternatives, such as the Wilcoxon signed-rank test or the Mann–Whitney U test, will be employed to analyze the data.

The presentation of the authors’ initial experience with CS serves as an effort to promote the use of CS in engineering, specifically in MRI hardware development. While CS is not widely used in the engineering domain, it is a popular and extensively applied format in biology, ecology and environmental sciences, where it is mostly used for collecting and classifying large amounts of data^35^. Here, an alternative approach was adopted that can be classified as collaborative^36^, meaning that the citizen scientists refine the research design and activities of a project generally designed by professional researchers. For this approach, the compact group of 24 upper secondary level students was perfectly suited. The students were mature enough to understand the scientific context and their tasks related to the project, making significant contributions to the developed questionnaire and the clinical study in general, and profiting from the added-value activities in a direct and sustainable way, as discussed below. The authors are convinced that the collaborative CS approach is applicable in a variety of engineering, natural science and medicine research projects. An example would be the assembly of simple motion sensors^37^ according to a construction manual—a task suitable for students of secondary technical schools—which could then be used in multi-center studies, if available in high quantity. Another example would be contributing to the investigation of patient perception of clinical workflows and patient communication strategies.

The research team consisting of full-time MRI scientists got a chance to interact with the “real world” on their research topic, i.e. with students who are not experienced in the field. The project format allowed them to gain experience in establishing a collaboration between scientists and other groups of society, in fostering community engagement and in democratizing access to research resources and data. Further, during preparation of the CS event content and material, researchers were invited to think “out of the box” and put a strong focus on didactic considerations rather than scientific content which will certainly also be beneficial for lectures or talks in the university environment. In many scientific projects, communication and dissemination activities play an important role and even though they often constitute a separate work package, the added benefit is not obvious to many researchers. In the authors’ opinion, CS initiatives are a purposeful and sustainable way of planning outreach activities. Not only do citizen scientists personally benefit but also provide the researchers with valuable input.

The student citizen scientists gained first-hand insight into research and the associated work. The initiative has a strong multidisciplinary orientation and combines the STEM-domain with medicine and humanistic elements. This way, students with widely differing interests could be reached and enthused about science, as confirmed by the involved teachers in concluding discussions and by the fact that three (female) students of the group registered for summer internships at the research institution. Potentially also having mostly female researchers involved in the project incited a large interest in female students. A stronger integration of CS projects in the school curriculum could further increase the educational outcome, but would require careful long-term planning that should ideally be completed before the beginning of the school year, so that the project can be accorded with class schedules and teacher assignments. However, this is hardly compatible with highly dynamic research settings. The discussion session for refining the questionnaire benefited from the fact that the students knew each other very well and, therefore, were very engaged in the discussion. It felt like the communication barrier between students and researchers was non-existent and both groups were at ease and free in expressing themselves. In this regard, the kick-off meeting and the program on-site helped to get familiar on a personal and scientific level. On the other hand, a more heterogeneous group of citizen scientists may have resulted in even more and/or other modifications of the developed questionnaire. In this regard, target group selection can be identified as a limitation of the presented CS initiative, as the students are not matched in age and educational background to the patient population in the “ModFlex” study, resulting in potential bias.

In the future, other CS events could be set up to provide a more inclusive questionnaire, e.g. to translate the items to other languages with the help of native speakers. A video with sign language and audio recording of the questionnaire could be prepared in a second step. Another approach would be to expand the questionnaire and get radiographers’ feedback regarding their experience with flexible coils. This could address questions such as usability, i.e. easier handling of the coils due to the lightweight design, faster coil positioning, improved patient compliance, lower patient drop-out rate, and faster overall workflow. Investigations in this direction will be launched with a small sub-study measuring MR imaging setup times with the novel flexible coils compared to the current clinical standard.

The concept of CS could also be exploited in the field of MR RF hardware beyond the aspect of patient comfort. For example, the development of flexible RF coils could benefit from the inclusion of textile designers and tailors as citizen scientists in the early stages of coil design. Their task could consist in bringing up innovative drafts optimizing the close-fitting design and comfort of the coil, or in assisting technical staff with the assembly during the production stage.

## Conclusions

This article reports the organization of a citizen science initiative with a group of students at the upper secondary school level who successfully contributed to establishing a questionnaire that can be used to evaluate the influence of RF coil design on patient perception of MRI examinations. This questionnaire will be directly employed in an ongoing clinical study and is made available in German and English to be used and/or adapted by others. The students’ involvement in the questionnaire was preceded by dedicated activities in CS events. These activities helped them to empathize with MRI patients, increased their understanding of healthcare technology and fostered a positive attitude towards science in general.

## Methods

### Ethics declarations

The presented study is conducted in accordance with the Declaration of Helsinki, and approved by the Ethics Committee of the Medical University of Vienna (2137/2021, date of approval: 16.03.2022). Informed consent was obtained from all subjects involved in the study. The investigated novel flexible RF coils are approved by national authorities under the Medical Device Regulation 2017/745 Article 62. Consent to publish the photographs shown in Fig. 2 in an online open access publication was obtained from all involved participants.

### Clinical study context

The “ModFlex” study is designed as a prospective mono-center cross-over trial, with the null hypothesis that there is no measurable difference between the investigated devices and reference products. The alternative hypotheses are that the investigated devices provide higher signal-to-noise ratio (endpoint 1) and higher ratings in a visual grading characteristics analysis^38^ (endpoint 2) than reference products. The proposed questionnaire on comfort during MRI exams aims to extend these alternative hypotheses: The MR imaging setup with flexible coils provides increased patient comfort (endpoint 3) and reduces pain (endpoint 4) in comparison to reference products.

The interventions in this study consist of two MRI exams. Each patient or volunteer will undergo one exam using the novel flexible RF coils and one using the standard reference product. After each measurement, the volunteer or patient will be given the same questionnaire to assess their physical and psychological well-being before, during and after the exam. To avoid bias, the order of the two measurements will be randomized.

The “ModFlex” study foresees a total of 108 subjects: 58 patients (female) with suspicious abnormalities in the breast and 50 healthy volunteers (male and female) from 19 to 80 years old and free of MRI contraindications. All included subjects should be able to understand and speak German. To date, 16 volunteers and 10 patients have already been measured. Preliminary sample size calculations for the questionnaire integration in this clinical study revealed that the remaining 82 potential participants are largely enough considering the calculated number of 34 required subjects to get a statistically significant outcome, assuming a significance level (α) of 0.05, an effect size of 0.5, and statistical power of 0.8, together with a 20% estimated dropout rate. A sub-analysis including only the remaining 48 breast patients will also yield a significant outcome according to sample size estimations.

### Questionnaire development

The following aspects were taken into account when designing the pre-CS version of the questionnaire: The time taken to fill the questionnaire should be relatively short (i.e., on average less than 5 min to respond to all questions). The language of the questionnaire was set to German according to the study context, with an English translation provided for the final version. Simple language should be used, since study participants are not necessarily German native speakers and have different educational backgrounds. Uncomplicated wording was considered essential to avoid obstacles or misunderstanding in completing the questionnaire and to minimize the dropout rate. As the focus lies on coil- and setup-related comfort evaluation, the subject should be asked for example, if lying still was easily possible, if repeated measurements or a longer measurement time would have been acceptable, how comfortable the lying position was, or if they were in pain. A chronological order of questionnaire items, i.e. in before, during and after the examination, should be followed to assess the temporal occurrence of discomfort, pain and stress. Subheadings in the questionnaire should make this classification clear to the participants and avoid confusion, e.g. if an item is repeated (to determine pre- vs. post- examination changes). Furthermore, to cover other factors, which could affect the subject’s perception of the measurement, they should be asked about their general well-being before the measurement, and if the loud noise during the measurement or small bore of the MRI scanner caused stress. The purpose of these neither setup- nor coil-related questions is to eventually subdivide the subjects into two groups for data analysis, i.e. those who were rather anxious or nervous in general (i.e., even before the exam) and those being rather calm. This could allow further insight in how the MRI exams using different coils were perceived depending on the general mindset and to control outliers, i.e. to check for drastic inconsistencies in the responses.

The resulting pre-CS version of the questionnaire consisted of a short introductory text, 18 items using a 7-point Likert scale, commonly used in social science surveys^39,40^, and two open-ended questions of which one proposes response options.

The pre-CS version was tested, reviewed and revised in plenum with the citizen scientists. In the follow-up phase of the CS initiative the feedback for the questionnaire provided by the students was analyzed, and three members of the research team reviewed the suggestions to decide which changes would be implemented. Decisions were mainly based on previous experience with patient MRI measurements. The questionnaires filled out by the students were collected and used as pilot data to define and set up a pipeline for the statistical analysis and visualization of the prospective results of the actual clinical study.

## Supplementary Information

Supplementary Information 1.

Supplementary Information 2.

## Data Availability

Except for the questionnaires included in Supplementary Data S1 and S2, no new data were created or analyzed in this study. Data sharing is not applicable to this article.
