# Supplementary material for: Citizen science approach to assessing patient perception of MRI with flexible radiofrequency coils
Source: Sci Rep. 2024 Feb 2;14:2811. doi: 10.1038/s41598-024-53364-x (PMC10837436; doi:10.1038/s41598-024-53364-x)
Supplement: Supplementary file 1 — Supplementary Information 1. [file 41598_2024_53364_MOESM1_ESM.pdf]

Fragebogen zur MRT-Untersuchung

LiebeR StudienteilnehmerIn, helfen Sie uns, die MRT-Forschung einen Schritt weiterzubringen, indem Sie diesen Fragebogen ehrlich beantworten. Alle Angaben werden anonym behandelt.

Kreuzen Sie bitte pro Frage auf der Skala von 1 bis 7 die für Sie zutreffende Antwort an.

stimme überhaupt nicht zu	stimme nicht zu	stimme eher nicht zu	weder noch/ neutral	stimme eher zu	stimme zu	stimme voll und ganz zu
1	2	3	4	5	6	7

Vor der Untersuchung (im Warteraum, in der Umkleide, beim Gespräch mit der Ärztin/dem Arzt):

1. Ich war aufgeregt, unruhig oder nervös.

stimme überhaupt nicht zu ◯ 1 ◯ 2 ◯ 3 ◯ 4 ◯ 5 ◯ 6 ◯ 7 stimme voll und ganz zu

2. Ich hatte Angst.

stimme überhaupt nicht zu ◯ 1 ◯ 2 ◯ 3 ◯ 4 ◯ 5 ◯ 6 ◯ 7 stimme voll und ganz zu

3. Ich habe mich wohl gefühlt.

stimme überhaupt nicht zu ◯ 1 ◯ 2 ◯ 3 ◯ 4 ◯ 5 ◯ 6 ◯ 7 stimme voll und ganz zu

Während der Vorbereitung und Untersuchung im Scannerraum:

4. Ich habe es umständlich oder körperlich anstrengend gefunden, die Liegeposition für die Untersuchung einzunehmen.

stimme überhaupt nicht zu ◯ 1 ◯ 2 ◯ 3 ◯ 4 ◯ 5 ◯ 6 ◯ 7 stimme voll und ganz zu

5. Ich habe es als unangenehm empfunden, dass manche Körperstellen nackt waren.

stimme überhaupt nicht zu ◯ 1 ◯ 2 ◯ 3 ◯ 4 ◯ 5 ◯ 6 ◯ 7 stimme voll und ganz zu

6. Ich habe mich wohl gefühlt.

stimme überhaupt nicht zu ◯ 1 ◯ 2 ◯ 3 ◯ 4 ◯ 5 ◯ 6 ◯ 7 stimme voll und ganz zu

7. Ich hatte Angst, in der engen Röhre zu liegen.

stimme überhaupt nicht zu ◯ 1 ◯ 2 ◯ 3 ◯ 4 ◯ 5 ◯ 6 ◯ 7 stimme voll und ganz zu

8. Es ist mir schwer gefallen, ruhig liegen zu bleiben (Ich hätte gerne Arme/Beine/Kopf bewegt).

stimme überhaupt nicht zu ◯ 1 ◯ 2 ◯ 3 ◯ 4 ◯ 5 ◯ 6 ◯ 7 stimme voll und ganz zu

9. Die lauten Geräusche haben mich verunsichert oder mir große Sorgen bereitet.

stimme überhaupt nicht zu ◯ 1 ◯ 2 ◯ 3 ◯ 4 ◯ 5 ◯ 6 ◯ 7 stimme voll und ganz zu

10. Ich habe die Liegeposition als bequem empfunden.

stimme überhaupt nicht zu ◯ 1 ◯ 2 ◯ 3 ◯ 4 ◯ 5 ◯ 6 ◯ 7 stimme voll und ganz zu

stimme überhaupt nicht zu	stimme nicht zu	stimme eher nicht zu	weder noch/ neutral	stimme eher zu	stimme zu	stimme voll und ganz zu
1	2	3	4	5	6	7

11. Ich hätte die Untersuchung am liebsten nach ein paar Minuten abgebrochen.

stimme überhaupt nicht zu ◯ 1 ◯ 2 ◯ 3 ◯ 4 ◯ 5 ◯ 6 ◯ 7 stimme voll und ganz zu

12. Ich hatte ein Hitzegefühl.

stimme überhaupt nicht zu ◯ 1 ◯ 2 ◯ 3 ◯ 4 ◯ 5 ◯ 6 ◯ 7 stimme voll und ganz zu

13. Ich wollte Polster, Kabel oder Geräte anders hinlegen.

stimme überhaupt nicht zu ◯ 1 ◯ 2 ◯ 3 ◯ 4 ◯ 5 ◯ 6 ◯ 7 stimme voll und ganz zu

14. Ich empfand während der Untersuchung Schmerzen.

stimme überhaupt nicht zu ◯ 1 ◯ 2 ◯ 3 ◯ 4 ◯ 5 ◯ 6 ◯ 7 stimme voll und ganz zu

Genauere Beschreibung der Schmerzen:

◯ Druckstellen ◯ Hitze ◯ Liegeposition ◯ Übelkeit ◯ Einschlafen der Arme/Beine

◯ Anderes: ____

Nach der Untersuchung:

15. Ich hätte noch weitere 15 Minuten aushalten können.

stimme überhaupt nicht zu ◯ 1 ◯ 2 ◯ 3 ◯ 4 ◯ 5 ◯ 6 ◯ 7 stimme voll und ganz zu

16. Ich würde einer Freundin bzw. einem Freund diese Untersuchung als sehr unangenehm beschreiben.

stimme überhaupt nicht zu ◯ 1 ◯ 2 ◯ 3 ◯ 4 ◯ 5 ◯ 6 ◯ 7 stimme voll und ganz zu

17. Ich würde es schlimm finden, die Untersuchung wiederholen zu müssen.

stimme überhaupt nicht zu ◯ 1 ◯ 2 ◯ 3 ◯ 4 ◯ 5 ◯ 6 ◯ 7 stimme voll und ganz zu

18. Unangenehme Druckstellen von der Untersuchung waren auch danach noch spürbar.

stimme überhaupt nicht zu ◯ 1 ◯ 2 ◯ 3 ◯ 4 ◯ 5 ◯ 6 ◯ 7 stimme voll und ganz zu

Was hat Sie noch beschäftigt?

____

____

Vielen Dank für Ihre Teilnahme!

Vom medizinischen Personal auszufüllen:

Code: ______________________________ ◯ MA ◯ MB ◯ R____

◯ Gruppe 1 – technisch: ◯ Hals ◯ Sprunggelenk ◯ Wirbelsäule ◯ Hüfte

◯ Gruppe 2 – Brust technisch ◯ Gruppe 3 – Brust klinisch: ◯ supine ◯ prone
