# Supplementary material for: Citizen science approach to assessing patient perception of MRI with flexible radiofrequency coils
Source: Sci Rep. 2024 Feb 2;14:2811. doi: 10.1038/s41598-024-53364-x (PMC10837436; doi:10.1038/s41598-024-53364-x)
Supplement: Supplementary file 2 — Supplementary Information 2. [file 41598_2024_53364_MOESM2_ESM.pdf]

MRI examination questionnaire

Dear study participant, please help us take MRI research one step further by answering this questionnaire honestly. All information will be treated anonymously.

For each question, please mark the answer that corresponds best on the scale from 1 to 7.

strongly disagree	disagree	somewhat disagree	neither agree nor disagree/ neutral	somewhat agree	agree	strongly agree
1	2	3	4	5	6	7

Before the examination (in the waiting room, in the changing room, when talking to the doctor):

1. I was anxious, agitated, or nervous.

strongly disagree ◯ 1 ◯ 2 ◯ 3 ◯ 4 ◯ 5 ◯ 6 ◯ 7 strongly agree

2. I was scared.

strongly disagree ◯ 1 ◯ 2 ◯ 3 ◯ 4 ◯ 5 ◯ 6 ◯ 7 strongly agree

3. I felt comfortable.

strongly disagree ◯ 1 ◯ 2 ◯ 3 ◯ 4 ◯ 5 ◯ 6 ◯ 7 strongly agree

During preparation and examination in the scanner room:

4. I found it cumbersome or physically demanding to assume the lying position for the examination.

strongly disagree ◯ 1 ◯ 2 ◯ 3 ◯ 4 ◯ 5 ◯ 6 ◯ 7 strongly agree

5. I found it unpleasant that some parts of my body were naked.

strongly disagree ◯ 1 ◯ 2 ◯ 3 ◯ 4 ◯ 5 ◯ 6 ◯ 7 strongly agree

6. I felt comfortable.

strongly disagree ◯ 1 ◯ 2 ◯ 3 ◯ 4 ◯ 5 ◯ 6 ◯ 7 strongly agree

7. I was afraid to lie in the narrow bore.

strongly disagree ◯ 1 ◯ 2 ◯ 3 ◯ 4 ◯ 5 ◯ 6 ◯ 7 strongly agree

8. I found it hard to lie still (I wanted to move my arms/legs/head).

strongly disagree ◯ 1 ◯ 2 ◯ 3 ◯ 4 ◯ 5 ◯ 6 ◯ 7 strongly agree

9. The loud noises strongly distressed me.

strongly disagree ◯ 1 ◯ 2 ◯ 3 ◯ 4 ◯ 5 ◯ 6 ◯ 7 strongly agree

10. I found the lying position comfortable.

strongly disagree ◯ 1 ◯ 2 ◯ 3 ◯ 4 ◯ 5 ◯ 6 ◯ 7 strongly agree

strongly disagree	disagree	somewhat disagree	neither agree nor disagree/ neutral	somewhat agree	agree	strongly agree
1	2	3	4	5	6	7

11. I would have preferred to stop the examination after a few minutes.

strongly disagree ◯ 1 ◯ 2 ◯ 3 ◯ 4 ◯ 5 ◯ 6 ◯ 7 strongly agree

12. I felt a sensation of heat.

strongly disagree ◯ 1 ◯ 2 ◯ 3 ◯ 4 ◯ 5 ◯ 6 ◯ 7 strongly agree

13. I felt the need to adjust the position of pillows, cables or devices.

strongly disagree ◯ 1 ◯ 2 ◯ 3 ◯ 4 ◯ 5 ◯ 6 ◯ 7 strongly agree

14. I was in pain during the examination.

strongly disagree ◯ 1 ◯ 2 ◯ 3 ◯ 4 ◯ 5 ◯ 6 ◯ 7 strongly agree

Detailed description of the pain:

◯ Pressure points ◯ Heat ◯ Lying position ◯ Nausea ◯ Arms/legs falling asleep

◯ Other: ____

After the examination:

15. I could have lasted another 15 minutes.

strongly disagree ◯ 1 ◯ 2 ◯ 3 ◯ 4 ◯ 5 ◯ 6 ◯ 7 strongly agree

16. I would describe this examination to a friend as very unpleasant.

strongly disagree ◯ 1 ◯ 2 ◯ 3 ◯ 4 ◯ 5 ◯ 6 ◯ 7 strongly agree

17. I would hate to have to repeat the examination.

strongly disagree ◯ 1 ◯ 2 ◯ 3 ◯ 4 ◯ 5 ◯ 6 ◯ 7 strongly agree

18. Unpleasant pressure points from the examination were still noticeable afterwards.

strongly disagree ◯ 1 ◯ 2 ◯ 3 ◯ 4 ◯ 5 ◯ 6 ◯ 7 strongly agree

What else is on your mind?

____

____

Thank you for your participation!

To be completed by the medical staff:

Code: ______________________________ ◯ MA ◯ MB ◯ R____

◯ Group 1 – technical: ◯ Neck ◯ Ankle ◯ Spine ◯ Hip

◯ Group 2 – breast technical ◯ Group 3 – breast clinical: ◯ supine ◯ prone
